# The Relationship Between COVID-19 Disease Severity and Zonulin Levels

**DOI:** 10.7759/cureus.28255

**Published:** 2022-08-22

**Authors:** Muhammed Okuyucu, Demet Yalcin Kehribar, Mustafa Çapraz, Aylin Çapraz, Mustafa Arslan, Zülfinaz Betül Çelik, Büşra Usta, Asuman Birinci, Metin Ozgen

**Affiliations:** 1 Internal Medicine, Ondokuz Mayıs University Faculty of Medicine, Samsun, TUR; 2 Internal Medicine, Amasya University Sabuncuoğlu Şerefeddin Education and Research Hospital, Amasya, TUR; 3 Pulmonary Medicine, Amasya University Sabuncuoğlu Şerefeddin Education and Research Hospital, Amasya, TUR; 4 Infectious Diseases, Amasya University Sabuncuoğlu Şerefeddin Education and Research Hospital, Amasya, TUR; 5 Medical Biology, Samsun University Faculty of Medicine, Samsun, TUR; 6 Medical Microbiology, Ondokuz Mayıs University Faculty of Medicine, Samsun, TUR; 7 Rheumatology, Ondokuz Mayıs University Faculty of Medicine, Samsun, TUR

**Keywords:** covid patients, inflammatory markers, inflammation, covid-19, zonulin

## Abstract

Introduction

Zonulin is a protein that plays a role in the reversible regulation of epithelial permeability. As zonulin is released in large amounts into the intestinal lumen, it disrupts the integrity of the tight junctions and causes continuous migration of antigens to the submucosa. Consequently, it can trigger inflammatory processes and severe immune reactions. In severe cases, SARS-CoV-2 may have a major impact on the clinical manifestations of the disease by directly or indirectly affecting intestinal cells and triggering systemic inflammation. Therefore, our study aimed to investigate the role of one of the possible mediators, zonulin, in the severity of the COVID-19 infection.

Methods

Thirty COVID-19 patients and 35 healthy controls were included in the study. Blood samples were taken from the patients on the 1st, 4th, and 8th days of hospitalization. Serum zonulin levels were determined by enzyme-linked immunosorbent assay (ELISA). Complete blood count (white blood cell [WBC], neutrophil, lymphocyte, and platelet), biochemical parameters (serum lactic acid dehydrogenase [LDH], erythrocyte sedimentation rate [ESR], C-reactive protein [CRP], D-dimer, ferritin, fibrinogen levels) were determined and chronic systemic disease states of the patients were assessed.

Results

Serum zonulin levels were notably higher in the healthy control group compared to the patient group (p=0.003). Although there was an increase in the zonulin values by time in hospitalization, this rising was not significant. Conversely, ESR and CRP levels were significantly higher in the patient group (p<0.001). There was no significant difference between the two groups regarding gender, age, and WBC counts.

Conclusion

The serum zonulin levels of COVID-19 patients with the mild clinical course were lower than the healthy control group. Moreover, serum zonulin levels were not correlated with ESR, CRP, and other inflammation markers. Our results suggest that low serum zonulin levels in COVID-19 patients might represent a mild disease course.

## Introduction

At the end of 2019, a new type of coronavirus called SARS-CoV-2 spread rapidly all over the globe and caused the death of millions of people under the name of the COVID-19 pandemic. Although COVID-19 mainly causes infection in the upper and lower respiratory tract, it also affects many different systems such as the gastrointestinal and central nervous systems, and progress from mild to severe clinical course that can be fatal [1-3].

Studies on acute respiratory distress syndrome (ARDS) have demonstrated that intestinal barrier integrity is impaired, and microbial translocation and dysbiosis trigger inflammation and disease severity [4,5]. Although it mainly causes clinical symptoms with lung involvement, gastrointestinal complications are also frequently seen in COVID-19 infection [6]. Lamers et al. investigated that relationship and found that in severe COVID-19 infection, SARS-CoV-2 might have a significant effect on the clinical manifestations of the disease by directly or indirectly affecting intestinal cells [6]. However, it has not yet been fully clarified whether this effect of COVID-19 in the intestine is directly caused by infection or indirectly by systemic inflammation and cytokines.

Zonulin is a protein that can increase permeability, especially in the jejunum and ileum, and cause the opening in the tight junctions of the intestinal epithelium [7]. The zonulin protein is also called pre-haptoglobin (Hp-2), which is the pro-protein of haptoglobin-2. Zonulin is found in the structure of tight junctions (TJ) in the intestine, which is involved in the paracellular transition [8]. It is well-known that zonulin reversibly regulates intestinal permeability by modulating tight junctions and plays an essential role in the absorption of nutrients, transport, and clearance of microorganisms [9,10]. Moreover, zonulin inhibits bacterial colonization in the small intestine and is involved in innate immunity [11,12]. Bacteria in the intestinal lumen and gluten in foods are the two most important factors that trigger the release of zonulin into the intestinal lumen [13,14]. Zonulin, released in large amounts into the intestinal lumen, binds to the receptors on the apical surfaces of the intestinal epithelial cells, disrupting the integrity of the tight junctions and increasing the intestinal permeability, causing the continuous pass of antigens from the intestine to the submucosa. Consequently, remainings of digested food and bacteria that enter the circulation can trigger an intense reaction in the immune system, triggering the inflammatory processes [10,15].

Studies have demonstrated that the 10% of individuals with a genetic predisposition have increased intestinal permeability because of various environmental stimuli, and eventually, this may result in the formation of various metabolic, allergic, or autoimmune diseases, such as celiac disease, inflammatory bowel disease, rheumatoid arthritis, and type-1 diabetes [12,16,17].

Zonulin is thought to involve in the pathophysiology of many autoimmune, neurodegenerative, and tumoral diseases. The present study aimed to investigate the possible relationship between zonulin and mild COVID-19 infection. Moreover, it is aimed to contribute to the current knowledge by investigating whether zonulin can be used as a biomarker in determining the severity of the disease in COVID-19 infection, where there is no clear consensus on its pathophysiology and treatment yet.

## Materials and methods

Patients

Thirty COVID-19 cases hospitalized in Amasya Sabuncuoğlu Şerefeddin Training and Research Hospital Infectious Diseases Clinic were prospectively included in this study. Nasal and pharyngeal swab samples were used to determination of COVID-19 positivity (2019-nCoV qPCR Detection Kit, Bioeksen Bio-Speedy R&D Co, Ltd, Turkey). In addition, a control group with age- and sex-matched 35 healthy volunteers (community-based random selection) were involved in the study. This study was approved by the Ondokuz Mayıs University Clinical Research Ethics Committee (Approval number: OMU KAEK 2022/220) and signed consent forms were obtained from all volunteers after they were informed. Pregnant women, patients younger than 18, and patients with malignancy were not included in the study. Moreover, patients who did not have breathlessness and hypoxia, did not need intensive care unit and mechanical ventilation, had symptoms not longer than 3 days, and had mild symptoms such as nausea, anorexia, and fever were included.

On the 1st, 4th, and 8th days of hospitalization, complete blood count parameters (white blood cell [WBC], neutrophil, lymphocyte, and platelet), biochemical parameters (serum lactic acid dehydrogenase [LDH], C-reactive protein [CRP], erythrocyte sedimentation rate [ESR], D-dimer, ferritin, fibrinogen, and zonulin) and chronic systemic disease (hypertension, diabetes mellitus, Alzheimer's disease, coronary artery disease, rheumatoid arthritis) states were recorded.

Measurement of the serum zonulin levels by ELISA

The blood samples taken from the patient and control groups were immediately centrifuged at 3000xg for 15 minutes, the sera were separated, and then the serum samples were stored at -80˚C till the day of the experiments. The sera were used by thawing at room temperature on the day of the enzyme-linked immunosorbent assay (ELISA) assay. The zonulin levels in serum samples were determined using a commercial ELISA kit (AFG Bioscience, #EK710802, Northbrook, USA). All analyses were performed in accordance with the manufacturer's protocol.

Statistical analysis

All the descriptive statistics for the continuous variables were presented as mean and standard deviation (SD). The linear data were evaluated using the paired-sample T-test. Furthermore, Pearson’s correlation analysis was performed. All statistical analyzes were conducted using the Statistical Package for Social Sciences (SPSS) Version 22.0 (IBM Corp, Armonk, USA). P-values less than 0.05 were considered significant.

## Results

Twenty (57.1%) of the healthy control group and 19 (63.3%) of the patient group were female. The mean age of the healthy control group was 52.8±15.6 years, and the mean age of the patient group was 59.8±17.3 years. Demographic characteristics and routine biochemical analysis results of the healthy control group and patients are represented in Table 1. There was no significant difference between the two groups regarding gender, age, and WBC values. On the other hand, while ESR and CRP levels were notably higher in the patient group (p<0.001), zonulin levels were significantly higher in the healthy control group (p=0.003).

**Table 1:** Demographic and laboratory data comparison of the study groups WBC: white blood cell, ESR: erythrocyte sedimentation rate, CRP: C-reactive protein. Data presented as mean ± standard deviation.

	Range/Normal Values	Healthy Controls	COVID-19 Patients	p-value
Gender (Female/Male)		20/15	19/11	0.800
Age (Year)		52.8±15.6	59.8±17.3	0.079
WBC (10^3^/uL)	3.9-10.9	6.8±1.4	6.6±2.4	0.711
ESR (mm/h)	0-20	13.5±8.3	57.8±31.4	<0.001
CRP (mg/L)	0-5	5.1±5.6	70.7±68.7	<0.001
Zonulin (pg/mL)		809±833	355±181	0.003

The most common chronic systemic disease in the patients was hypertension (n:9, 30%). In the routine follow-ups of the patients, there was no general condition disorder or the need for intensive care in any of the patients. The distribution of patients according to their chronic systemic disease condition is presented in Table 2.

**Table 2:** The distribution of the patients according to their chronic systemic disease (CSD) state * More than one option has been selected.

CSD* n (%)	Hypertension	9 (30)
	Diabetes mellitus	5 (16.6)
	Benign prostatic hyperplasia	4 (13.3)
	Chronic obstructive pulmonary disease	3 (10)
	Alzheimer’s disease	2 (6.6)
	Coronary artery disease	1 (3.3)
	Rheumatoid arthritis	1 (3.3)

**Figure 1:**
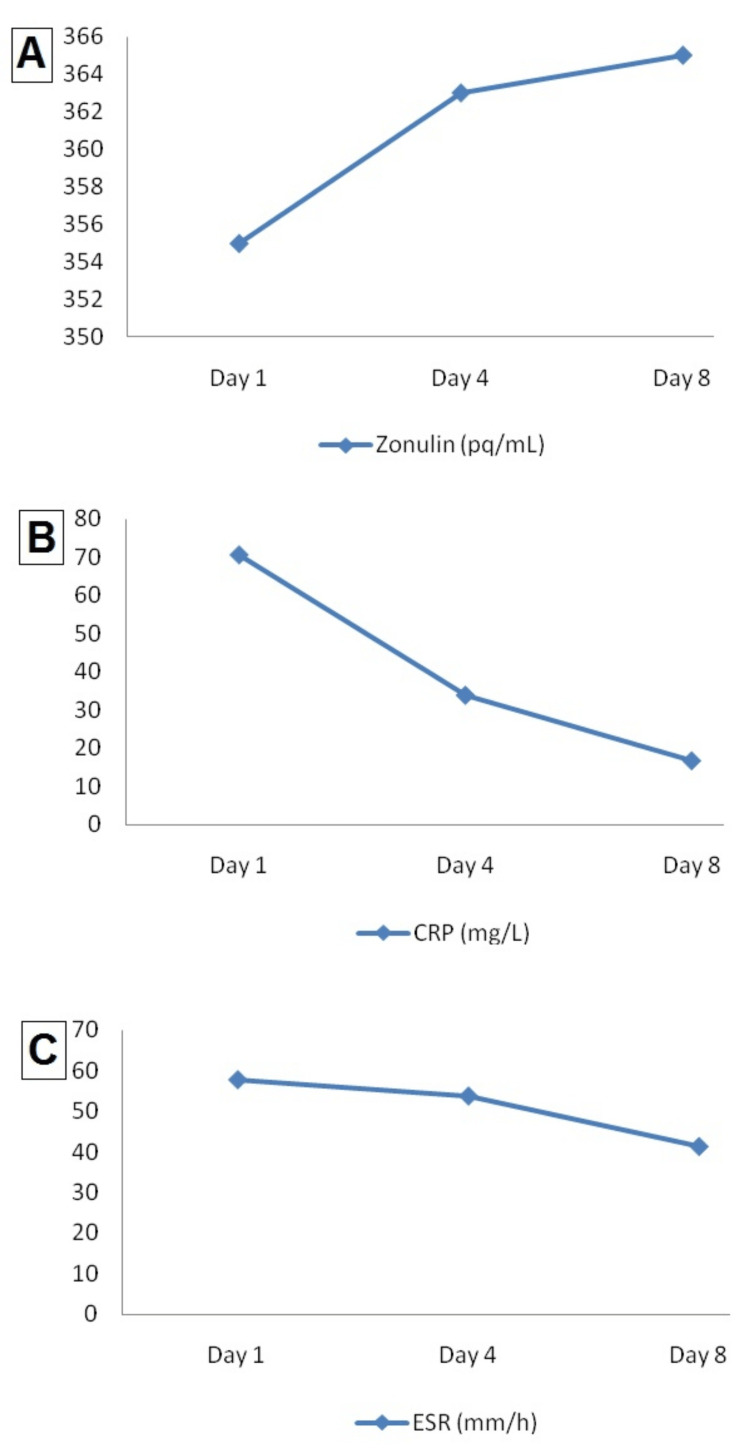
The graphs display the serum Zonulin (A) and CRP (B) levels and ESR (C) of COVID-19 patients on the 1st, 4th, and 8th days of hospitalization CRP: C-reactive protein, ESR: Erythrocyte sedimentation rate

Although there was an increase in the zonulin values of the patients over time, this rising was not statistically significant (p=0.604). Contrary to zonulin, ESR and CRP values decreased with clinical improvement in patients (p=0.024 and p=0.001, respectively). The laboratory data of the patients according to the 1st, 4th and 8th days are given in Table 3. There was no significant correlation between zonulin and other biochemical parameters. Correlation values between zonulin, CRP, and ESR, respectively, are r=-0.097, p=0.611 and r=-0.307, p=0.098 (Figure 1).

**Table 3:** The distribution of the laboratory data of the patients according to the analysis days WBC: white blood cell, LDH: lactate dehydrogenase, ESR: erythrocyte sedimentation rate, CRP: C-reactive protein. Data presented as mean ± standard deviation.

	Range/Normal Values	Day 1	Day 4	Day 8	p-value
WBC (10^3^/uL)	3.9-10.9	6.6±2.4	10.4±4.6	10.4±4.0	<0.001
Neutrophil (10^3^/uL)	1.8-6.9	4.9±2.3	8.6±4.5	8.6±3.5	<0.001
Lymphocyte (10^3^/uL)	1.2-3.3	1.2±0.6	0.9±0.5	1.1±0.8	0.541
Platelet (10^3^/uL)	166-308	200±54	259±71	304±105	<0.001
LDH (U/L)	135-214	325±114	287±100	274±80	0.024
Fibrinogen	200-400	512±120	447±91	360±103	<0.001
Ferritin (ng/mL)	21-274	308±347	282±261	371±366	0.260
D-dimer		1.40±2.74	0.81±1.29	0.88±1.24	0.280
ESR (mm/h)	0-20	57.8±31.4	53.8±28.4	41.4±23.4	0.024
CRP (mg/L)	0-5	70.6±68.7	33.8±36.8	16.6±29.9	0.001
Zonulin (pg/mL)		355±181	363±205	365±197	0.604

## Discussion

In our study, zonulin levels on the 1st, 4th, and 8th days were investigated in the sera of patients diagnosed with COVID-19 who had mild clinical course. Serum zonulin levels of COVID-19 patients were lower than the healthy control group. Moreover, serum zonulin levels were not correlated with ESR, CRP, and other inflammation markers.

Kılıç et al. found that zonulin levels were significantly elevated in multisystem inflammatory syndrome (MIS-C) patients with COVID-19 than in COVID-19 patients without MIS-C and the healthy control group. Based on this, they suggested that elevated zonulin levels may exacerbate inflammation in COVID-19 patients with MIS-C syndrome by increasing intestinal permeability [18]. While four of the 19 children diagnosed with COVID-19 who participated in their study were hospitalized, one child needed intensive care, and one died. However, the patients included in that study were children, and 31.4% of the patients had a more severe course, which is completely different patient profile with our study. Even though the results of the pairwise comparison of zonulin levels in COVID-19 patients with the healthy control group were not given in their study, it was stated that zonulin might play a role in the pathogenesis of COVID-19, and MIS-C syndrome.

Giron et al. investigated the relevance between intestinal permeability and the severity of COVID-19. They found that zonulin levels in moderate and severe COVID-19 patients increased notably compared to mild COVID-19 and healthy control groups [19]. However, in their study, it was not stated on which day of the disease the blood zonulin levels of COVID-19 patients were examined. In addition, since consecutive zonulin assessments were not performed, no correlation could be established between the clinical course of the disease and the zonulin level. In our study, like moderate patient groups of Giron et al., COVID-19 patients were evaluated, and serum zonulin levels of these patients were lower than the healthy control group.

Haptoglobin is an acute-phase protein that binds intravascular free hemoglobin (Hb), prevents oxidative stress caused by free Hb, and is a marker of general inflammation [20]. In humans, haptoglobin exists in two genetic variants as haptoglobin-1 and haptoglobin-2 [21]. In the studies of Delanghe et al and Kasvosve et al, it was stated that the prognosis of chronic infections such as HIV and tuberculosis is worse in individuals with the Hp2-2 genotype [22,23]. Studies have also shown that Hp-2 is associated with autoimmune disorders, epilepsy, and increased diabetic complications [24,25]. Moreover, COVID-19 infection causes a cytokine storm in some individuals thus causing the disease to have a more severe course. Therefore, it is considered that investigating the role of the Hp-2 genotype in the severe course of COVID-19 infection may help illuminate the disease's pathogenesis. At the onset of inflammation, pre-haptoglobin (zonulin) transforms into haptoglobin. Therefore, while pre-haptoglobin levels decrease, haptoglobin levels increase as an acute phase reactant [26]. However, it has been reported that haptoglobin level decreases in conditions such as adult-onset Still's disease and MIS-C, where inflammation is severe and haptoglobin precursor zonulin increases [27]. This decrease in haptoglobin level may be due to the inhibition of the conversion of pre-haptoglobin to haptoglobin in severe inflammation. As a result, it is considered that elevated zonulin increases intestinal permeability excessively in cases where inflammation is very severe, such as MIS-C and severe COVID-19 infection, which is one of the essential mechanisms effective in the pathogenesis [10]. In our study, the low serum zonulin levels in COVID-19 patients with a moderate clinical course can be explained by the conversion of pre-haptoglobin to haptoglobin in cases of non-severe inflammation.

The fact that the patient profile included in this study was only COVID-19 patients with mild symptoms may be a limitation of the study. Including patients with severe COVID-19 could have strengthened the study results. In addition, the blood zonulin levels of the patients were determined on the 1st, 4th, and 8th days, and it would be better to check them after the convalescent period. Moreover, measuring the haptoglobin levels as well as zonulin levels could help us better understand the mechanism of zonulin.

## Conclusions

In conclusion, the serum zonulin levels were found to be lower in COVID-19 patients compared to healthy controls. Considering the data of previous studies, the fact that zonulin levels do not increase in COVID-19 patients may be a sign that the disease will be milder. This can be explained by the conversion of pre-haptoglobin to haptoglobin in cases of non-severe inflammation. In severe inflammatory conditions such as MIS-C, this conversion is inhibited, and zonulin levels increase. New studies are needed to elucidate the role of zonulin in patients with COVID-19 and diseases with severe inflammatory conditions.
